# Editorial: Women in plant science - redox biology of plant abiotic stress 2022

**DOI:** 10.3389/fpls.2023.1236150

**Published:** 2023-06-22

**Authors:** María C. Romero-Puertas, Sabine Lüthje, Ana Zabalza, Laura de Gara, Christine H. Foyer

**Affiliations:** ^1^ Department of Biochemistry, Cell and Molecular Biology of Plants, Experimental Station of Zaidín, Spanish National Research Council (EEZ-CSIC), Granada, Spain; ^2^ Biodiversity of Crop Plants, Universität Hamburg, Institute of Plant Science and Microbiology, Oxidative Stress and Plant Proteomics Group, Hamburg, Germany; ^3^ Institute for Multidisciplinary Research in Applied Biology (IMAB), Universidad Pública de Navarra (UPNA), Pamplona, Spain; ^4^ Department of Science and Technology for Sustainable Development and Human Health, Università Campus Bio-Medico di Roma, Rome, Italy; ^5^ Department of Biosciences, University of Birmingham, Birmingham, United Kingdom

**Keywords:** redox biology, plants, abiotic stress, hydrogen peroxide, nitric oxide

## Introduction

Plants sustain all life on Earth. They are the basis for all the food that we eat, the oxygen that we breathe and our fossil fuels. They will also be essential in reducing atmospheric CO_2_ levels to combat anthropogenic CO_2_ release and sustaining the soil carbon sink. Plants are also extremely resilient, rapidly adjusting to continuous environmental changes. Rapid or acute changes in environmental conditions may however cause stress that results in complex responses and specific signalling local and long-distance pathways that underpin survival. These pathways include numerous signalling molecules, such as reactive oxygen, nitrogen and sulphur species (ROS/RNS/RSS). These function together with ions and phytohormones to adjust metabolism to prevailing environmental conditions. Some signalling molecules are able to generate protein post-translational modifications (PTMs) on target proteins, that affect their stability, localization, activity, binding to other proteins, etc. For example, redox PTMs involve reversible changes to cysteine thiol groups. These redox changes form the basis of hydrogen peroxide signalling and “Redox Biology”. Understanding the mechanisms underlying plant responses to changing conditions, particularly environmental pollutants is one of the most challenging tasks in plant science. Understanding the regulation of these processes will allow better targeting of approaches to improve plant stress adaptation and sustainable yield increases.

The contributions and achievements of women are essential to the 2030 Agenda for Sustainable Development. The aim of this Research Topic was to bring together the latest advances and related information from researchers working in the field of redox biology, particularly in abiotic stress tolerance. We celebrate the success of female scientists and seek to empower and encourage their future work. Female scientists make significant contribution to all fields of plant science. Examples of their work is presented in this Research Topic, in which all the articles have female corresponding authors. These papers not only highlight the importance of redox biology in plant responses to a diverse range of abiotic stresses, but they also present original advances in knowledge, approaches, and methods with potential applications in agriculture. Three reviews in this Research Topic summarise current concepts in redox biology, particularly PTMs and the role of oxidative metabolism in plant responses to abiotic stress.

## Original research

Three of the original manuscripts presented in this Research Topic are related to the responses of plants to extremes of temperature. Unpredictable temperature extremes such as heatwaves that are linked to climate change, pose serious threat to plant development and yields. Jindal et al. identified the role of type-A arabidopsis response regulators (ARR), which are competitors of the main components of cytokinins (CKs) signalling, the transcription factors type-B ARRs, in plant response to heat stress (HSR). Type-A ARRs negatively mediate HSR in arabidopsis (*Arabidopsis thaliana* (L.) Heynh.) and mutants lacking functional ARRs experience a priming state increasing their thermotolerance to elevated temperatures, showing proteomic changes similar to that of a heat acclimation. Interestingly, type-A ARR mutants showed an enhancement of low molecular weight antioxidants such as ascorbate and glutathione. GENOMES UNCOUPLED 1 (GUN1) is a key protein for retrograde signalling from chloroplast to the nucleus and it has been shown to be essential in plant thermotolerance in arabidopsis in the manuscript by Lasorella et al. *gun1* mutants fail to induce a transient oxidative burst after heat shock treatment, which seems to be essential for the activation of stress responsive genes during the recovery period. The analysis of different redox and reactive oxygen species (ROS)-related parameters in wt and *gun1* mutants after heat shock treatment and recovery period suggests a key role for tylacoidal ascorbate peroxidise in GUN1-dependent thermotolerance. Furthermore, iodoacetyl tandem mass tags (iodo-TMT)-proteomic analysis in cold acclimated rape (*Brassica napus* L.) after freezing stress resulted in the identification of 171 differentially sulfenylated proteins involving this redox-dependent post-translational modifications (PTM) in plant response to cold (Liangqian et al.). Interestingly, enzymes from the Calvin-Benson-Bassham cycle were sulfenylated after freezing affecting to the metabolite levels resulted from this pathway, involving again the chloroplasts in plant acclimation to cold stress. In fact, carbon fixation is a critical issue to manage climate crises. Woody plants like Norway spruce (*Picea abies* L. Karst.) are important carbon sinks. Photosynthetically fixed carbon is stored in plant cell walls that are comprised primarily of polysaccharides (Verbančič et al., 2018). Plant respiratory burst oxidase homologs (Rboh) are involved in abiotic stress response and cell wall modification by the production of ROS. The focus of the contribution by Nickolov et al. was on the regulation of gymnosperm Rboh biochemistry. They demonstrated the regulation of PaRBOH1-mediated ROS production in Norway spruce by Ca^2+^ binding and phosphorylation with a combination of in silico analysis, biochemistry and phosphoproteomics. On the other hand, herbicides still are the most commonly used mechanisms for weed control, which is a big challenge for crop protection. Eceiza et al. linked oxidative metabolism with high concentration of the herbicide nicosulfuron sensitivity in pigweed (*Amaranthus palmeri* S.Watson) plants. Oxidative stress however is not the only causes of plant death. Interestingly, most of the oxidative parameters analysed under control conditions were similar in sensitive and resistant populations showing that sensitivity is not due to different initial oxidative status.

## Technical advances

Allan et al. showed the development of a chip-based treatment technique paired with a reporter gene approach to measure cytosolic Ca^2+^ transients in arabidopsis in response to osmotic and salt stress. In contrast to published chip-based methods, the modification of the chip described here has the advantage to permit a more localized application of the treatment, a free choice of the direction of flow inside the chip and also a unilateral application of the test solution. This dual-flow RootChip (dfRC) could advance our understanding of how plants perceive and decode stress information in specific tissues at the cellular or subcellular levels. This is of relevance not only for stress research but also for other applications.

The results of the study were comparable to other studies using Ca2^+^ reporters on NaCl and osmotic stress (also cited in the references). There are also a number of studies using Ca2^+^ reporters on NaCl and osmotic stress (also cited in the references).

## Reviews

Although the contamination of the environment with nanoplastic is an emerging issue worldwide, until recently the attention has not been drawn to the effects of nanoplastics in plants. Ekner-Grzyb et al. revises up-to-date information with special emphasis on the oxidative response. Exposure to nanoplastics leads to increase in reactive oxygen species, and the altered nutrient photosynthesis levels result in hampered plants growth. In addition, nanoplastics alert plants physiology through modulation of genes expression on the transcriptomic level. The knowledge generated in recent years on redox post-translational modifications and their interplay in the acclimatization processes of plants is gathered in the review by Martí-Guillen et al.. The overproduction of molecular reactive species, mainly reactive oxygen, nitrogen and sulphur species, has been recorded after abiotic stresses, and they can modify proteins by PTMs. This process is proposed to not occur randomly but that the enzymatic activity can be modulated by the modification of critical amino acid residues. The review focuses on the redox-based post transcriptional modifications of the enzymes that participate in the main stress-related pathways, such as oxidative metabolism, primary metabolism, cell signalling events, and photosynthetic metabolism. Especial attention to nitric oxide, which promotes the first step in the redox state of the thiol groups from cysteines, so-called S-nitrosylation, is paid in the review by Mata-Pérez et al. This review collects significant and recent advances in NO-dependent PTMs, in particular, S-nitrosylation, in plant response to different abiotic stresses. Therefore, key results involving NO function in plant response to drought and salt stress, high and low temperature, mechanical wounding, heavy metals, hypoxia, UV radiation and ozone exposition are discussed.

## Author contributions

MR-P prepared the draft with SL, LG and AZ contributions, CF worked on it and then all the authors contributed with their suggestions to the final version.
